# Combined Exercise Therapy and Alternating Magnetic Field Therapy for Chemotherapy-Induced Peripheral Neuropathy in a Rectal Cancer Survivor: A Case Report

**DOI:** 10.7759/cureus.89262

**Published:** 2025-08-02

**Authors:** Keiichi Osaki, Shinichiro Morishita, Akiho Kamimura, Saki Yanai, Masayoshi Nakanishi

**Affiliations:** 1 Department of Rehabilitation, Panasonic Health Insurance Organization, Matsushita Memorial Hospital, Osaka, JPN; 2 Department of Physical Therapy, School of Health Sciences, Fukushima Medical University, Fukushima, JPN; 3 Department of Gastrointestinal Surgery, Panasonic Health Insurance Organization, Matsushita Memorial Hospital, Osaka, JPN

**Keywords:** alternating magnetic field therapy, cancer survivor, chemotherapy-induced peripheral neuropathy (cipn), exercise therapy, return-to-work support

## Abstract

Chemotherapy-induced peripheral neuropathy (CIPN) is a common and often persistent adverse effect of several anticancer agents, leading to impaired physical function and quality of life. Although exercise therapy and physical modalities such as transcutaneous electrical nerve stimulation have been reported to alleviate CIPN symptoms, the efficacy of alternating magnetic field therapy remains unclear. We here report a rectal cancer survivor who experienced difficulty returning to work due to persistent neuropathic symptoms of CIPN. A combination of exercise therapy and alternating magnetic field therapy improved the patient’s physical function and pain, ultimately enabling his return to work. This case highlights the potential utility of combining rehabilitation and alternating magnetic field therapy in CIPN management.

## Introduction

Chemotherapy-induced peripheral neuropathy (CIPN) is a common adverse effect associated with various chemotherapeutic agents, particularly taxanes, vinca alkaloids, and platinum-based drugs. Approximately 30-40% of patients treated with these agents develop CIPN [1]. The symptoms of CIPN predominantly present as sensory axonal neuropathy, often accompanied by mild-to-severe neuropathic pain. Beyond sensory deficits, CIPN may also affect motor and autonomic functions, potentially interfering with activities of daily living. Moreover, symptoms may worsen not only during chemotherapy but also after treatment completion, significantly impairing the patient’s quality of life (QOL) [1,2].

Currently, no established preventive or curative pharmacological treatments exist for CIPN, and the evidence supporting symptomatic interventions remains limited [2,3]. Although the evidence is still insufficient, several recent studies have suggested that exercise therapy may be effective in alleviating CIPN symptoms. In particular, aerobic exercise, resistance training, and core stability exercises have been reported to improve physical function, reduce neuropathic pain, and enhance QOL in patients with CIPN [3-5]. Similarly, electrical stimulation therapy has been reported to have a positive effect on pain relief, and combined interventions using both exercise and electrical stimulation have also been reported to be beneficial [5,6].

In contrast, no prior studies have reported the efficacy of alternating magnetic field (AMF) therapy for CIPN. AMF therapy is thought to modulate pain through descending inhibitory pathways and opioid systems, and has been reported to be effective in reducing puncture pain in dialysis patients, as well as fibromyalgia pain [7-9]. AMF therapy can be considered a relatively safe option for cancer patients due to its few contraindications; however, its efficacy for CIPN remains unclear.

This case report describes a colorectal cancer survivor who developed CIPN symptoms during chemotherapy and continued to experience them long after treatment completion, which made returning to work difficult. The symptoms were difficult to manage with pharmacological therapy alone. To support functional recovery toward returning to work, a physical therapy intervention was implemented. Based on previous studies reporting the benefits of exercise therapy for CIPN, the program included both exercise and AMF therapy. Considering the patient's prolonged numbness and pain, and in order to avoid the discomfort often associated with conventional electrical stimulation, AMF therapy was selected as an alternative modality. As a result, the patient showed improvement in pain and physical function and was ultimately able to return to work.

## Case presentation

Patient information

A male patient in his 50s, employed in a desk-based sales position, was diagnosed with rectal cancer at the Department of Gastrointestinal Surgery at our hospital, and subsequently underwent robot-assisted high anterior resection of the rectum. The pathological stage was classified as pStage III, with a TNM (tumor, node, and metastasis) classification of T4aN2aM0. His medical history included a diagnosis of depression 16 years prior. As adjuvant chemotherapy, eight cycles of CapeOX therapy were administered. At the first cycle, oxaliplatin was administered at a dose of 230 mg, based on the standard dose per body surface area. Two weeks after starting chemotherapy, he began to experience numbness in his distal extremities. From the third cycle, the dose was reduced to 190 mg due to worsening peripheral neuropathy. For the same reason, the dose was further reduced to 140 mg at the sixth cycle and to 110 mg at the seventh cycle. Oxaliplatin was discontinued at the eighth cycle. The cumulative dose of oxaliplatin was 1280 mg. Peripheral neuropathy was assessed according to the Common Terminology Criteria for Adverse Events (CTCAE) Version 5.0. It was classified as Grade 1 at the second cycle and progressed to Grade 2 approximately three months after the initiation of chemotherapy.

The patient’s numbness and pain persisted even after the completion of chemotherapy, leading to a relapse of depression and resulting in a leave of absence from work. As the symptoms continued to make returning to work difficult even 11 months after completing chemotherapy, he consulted with the cancer support center and, after discussing with his primary physician, a decision was made to initiate physical therapy.

At the start of rehabilitation, the patient was taking the following medications: sodium valproate (Depakene^®^ 200 mg); zolpidem tartrate (Myslee^®^ 5 mg); brotizolam (Lendormin^®^ 0.25 mg); perospirone hydrochloride (Lullan^®^ 4 mg); and duloxetine hydrochloride (Cymbalta^®^ 20 mg).

Duloxetine hydrochloride had been prescribed for depression and was taken continuously for a long period prior to the onset of CIPN. However, despite ongoing use of duloxetine, the patient developed CIPN symptoms, and even after the completion of chemotherapy, pain and numbness remained poorly controlled, making it difficult for the patient to return to work. Therefore, a referral for physical therapy was considered, with the goal of improving physical function and alleviating pain to support the patient's return to work. The interval between the end of treatment and the initiation of physical therapy was approximately 11 months after the completion of chemotherapy, and about one year after the final administration of oxaliplatin.

Physical therapy assessment

The patient's primary complaints related to difficulties in daily life were assessed through interviews, while numbness and pain were evaluated using the Numerical Rating Scale. Subjective symptoms were also assessed using the Patient Neurotoxicity Questionnaire (PNQ), for which formal permission for use in this study was obtained from the original author [10]. Physical function was evaluated through measurements of grip strength, pinch strength (tip, lateral, and palmar) [11], knee extension strength, six-minute walk test (6MWT) [12], and the Short Physical Performance Battery (SPPB) [13]. Grip strength and knee extension strength were measured in accordance with the methods used in previous studies [14]. Grip strength and knee extension strength were assessed according to previously established methods. Each measurement was performed twice on both sides, and the maximum value for each was recorded. Pinch strength was also measured based on prior studies, with three measurements taken for each side, and the highest value was recorded. Upper limb dysfunction and QOL were assessed using the Quick Disabilities of the Arm, Shoulder and Hand (QuickDASH) and the EuroQol 5-Dimension 5-Level (EQ-5D-5L) [15,16]. Formal permission to use the EQ-5D-5L was obtained (Registration ID: 75184). Evaluations were conducted at the start of rehabilitation and one, two, and three months thereafter.

Intervention and progress

**Figure 1:**
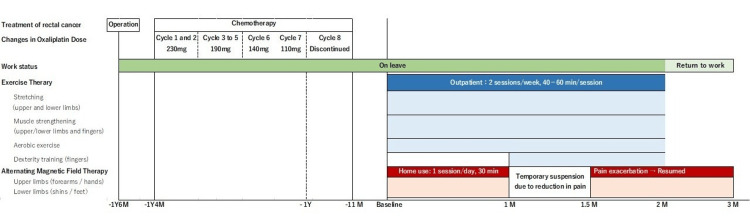
Rehabilitation interventions and timeline of progress Alternating magnetic field therapy was temporarily discontinued after one month due to a reduction in pain. However, it was resumed at 1.5 months following an exacerbation of pain, using the same settings as before.

An overview of the intervention is provided in Figure 1. The patient’s primary complaints included numbness and pain in the fingers, which made returning to desk work difficult, and severe fatigue following prolonged commutes. His PNQ scores for both sensory and motor symptoms were graded as D, indicating a clear impact on work and daily activities. Accordingly, we initiated exercise therapy aimed at facilitating his return to work, along with AMF therapy for pain control.

**Figure 2:**
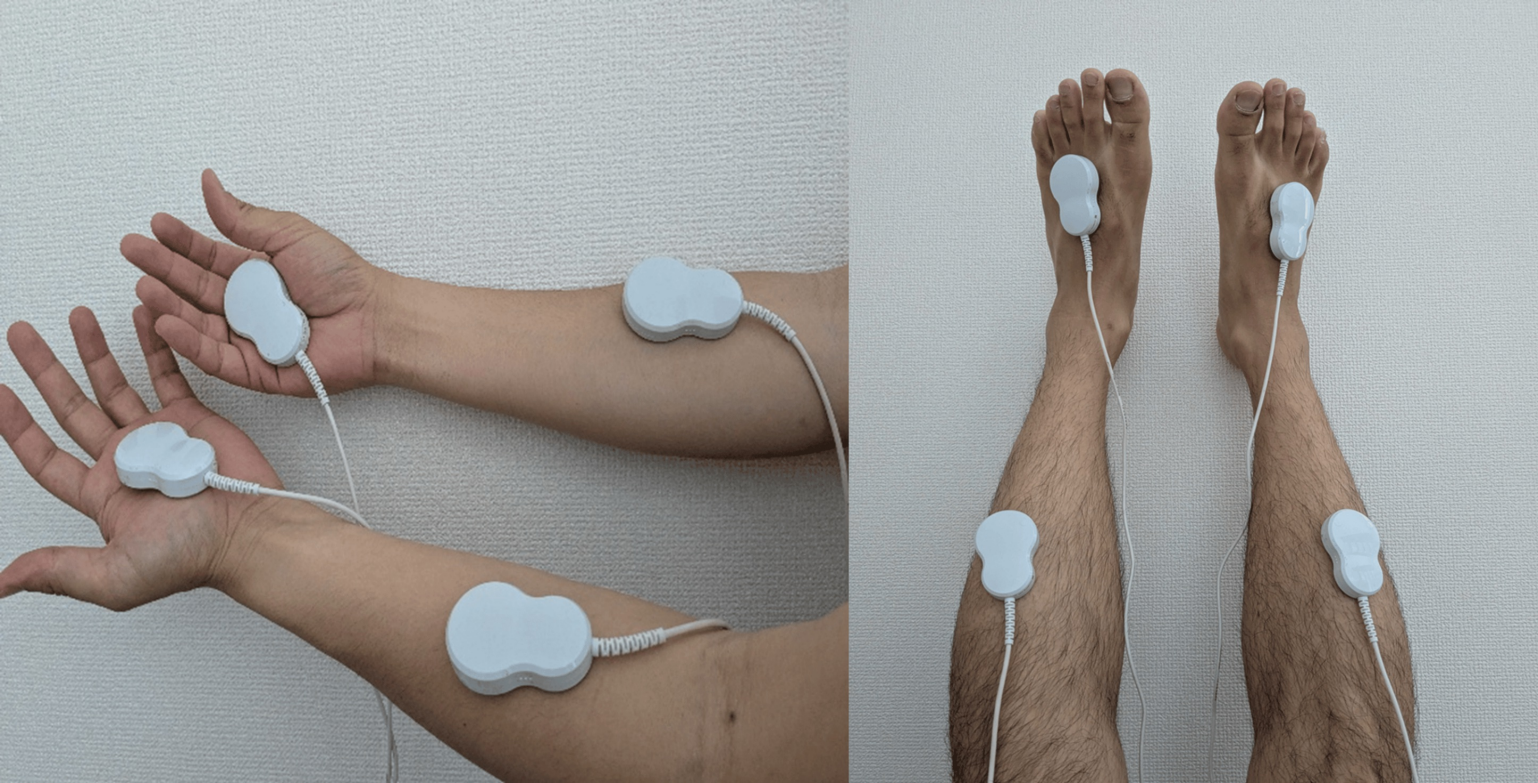
Application of alternating magnetic field therapy Irradiation pads were applied to the forearms and hands of both upper limbs, and to the shins and feet of both lower limbs. Each region (upper and lower limbs) received 30 minutes of irradiation per day.

Exercise therapy was administered twice a week (40-60 minutes per session) on an outpatient basis over two months. During the first month, the program focused on limb stretching, strengthening exercises (for upper and lower limbs and fingers), and aerobic exercise. Strength exercises were performed in sets of 10 repetitions, beginning with an intensity corresponding to 13 (“somewhat hard”) on the Borg Scale of Rating of Perceived Exertion, and progressively increased to a perceived intensity of 15 (“hard”) over time. A total of three to five sets were performed. Upper limb and finger exercises were conducted using resistance bands, hand grippers, and balls, while lower limb exercises primarily involved bodyweight training. Aerobic exercise was performed in 20-minute sessions at an intensity ranging from 11 (“light”) to 13 on the Borg scale. In the second month, pegboard training and computer-based dexterity exercises were added to enhance finger sensation. His occupation involved desk work, which required the use of a computer. Therefore, typing practice not only aimed to improve sensory function in the fingers but also served as essential preparation for returning to work. The therapy was completed when the patient returned to work at the end of the second month. AMF therapy was introduced concurrently with exercise therapy, using ait^®^ (Peace of Mind Co., Ltd., Kumamoto, Japan). When the start button is pressed, the device automatically generates two types of AMFs: one at 2 kHz and the other at 83.3 MHz, using coils located inside the irradiation pad. A usage time of at least 30 minutes per session is recommended, and the device is programmed to deliver a 30-minute treatment automatically. Since the total daily usage time is limited to a maximum of 2 hours, a 30-minute session was performed every day in this case. The therapy was applied daily at home to both the upper limbs (forearms and hands) and the lower limbs (shins and feet) (Figure 2). After one month, AMF therapy was temporarily suspended to evaluate its efficacy. However, due to an increase in pain, the therapy was resumed and continued until the end of the third month.

Outcomes

Physical function generally improved following exercise therapy. Although pinch strength and knee extension strength had slightly declined at the three-month follow-up, grip strength, 6MWT distance, and SPPB scores remained improved (Table 1).

**Table 1:** Changes in physical function 6MWT = 6-minute walk test; SPPB = Short Physical Performance Battery.

Measurement items	Baseline	1 month	2 months	3 months
Grip strength (right/left, kgf)	16.8/14.7	27.8/31.1	26.5/30.2	31.0/33.7
Pinch strength (right/left, kgf)				
Tip pinch	3.0/2.0	6.5/5.0	6.0/6.0	5.0/5.0
Lateral pinch	7.0/6.0	8.5/6.5	8.5/7.5	8.0/7.5
Palmar pinch	7.0/3.5	7.5/5.0	8.0/6.5	7.5/6.0
Knee extension strength (right/left, kgf/kg)	0.61/0.62	0.73/0.65	0.84/0.75	0.75/0.64
6MWT (m)	431	536	590	600
SPPB (points)	12	12	12	12
Balance test	Tandem stand possible	Tandem stand possible	Tandem stand possible	Tandem stand possible
Gait speed test (s)	3.53	3.21	2.64	2.29
Chair stand test (s)	6.54	6.82	5.52	5.08

At baseline, prior to the initiation of physical therapy, the patient reported bilateral numbness and pain in the distal parts of both the upper and lower limbs, specifically, distal to the forearms in the upper limbs and distal to the shins in the lower limbs. These symptoms were most prominent in the distal regions of both extremities, with numbness and pain perceived as equally intense on both sides. Forearm numbness resolved, and finger numbness was alleviated, although moderate residual symptoms persisted. Numbness in the lower limbs persisted in the lower legs, but improved in the feet, with moderate symptoms still present at three months. Hand pain at rest was markedly reduced, while exertional pain remained, and lower limb pain also persisted (Table 2).

**Table 2:** Changes of numbness and pain (Numerical Rating Scale) All scores are based on the Numerical Rating Scale (score is a 0-10 scale). The patient reported comparable symptoms bilaterally.

Symptom location	Baseline	1 month	2 months	3 months
Numbness				
Forearm	5	0	0	0
Palm to fingers	9	6	6	6
Lower leg	5	5	5	5
Foot	9	8	7	6
Pain (at rest/during movement）				
Palm to fingers	7/9	3/8	3/8	3/6
Foot	5/5	4/4	4/4	3/3

QuickDASH scores improved progressively from 59.1 to 43.2 over the three months. After returning to work, difficulties related to job performance were rated as “moderate,” while difficulties related to working hours were rated as “none.” Although EQ-5D-5L utility scores did not change substantially, pain/discomfort improved at a moderate level, while improvements were noted in the domains of usual activities and anxiety/depression (Table 3).

**Table 3:** Changes of EQ-5D-5L EQ-5D-5L = EuroQol 5-Dimension 5-Level. A utility index of 0 represents death, and a utility index of 1 represents perfect health.

EQ-5D-5L domain	Baseline	1 month	2 months	3 months
Utility index	0.616	0.558	0.688	0.576
Mobility	No problem	Slight problems	No problem	Slight problems
Self-care	Slight problems	Moderate problems	Slight problems	Slight problems
Usual activities	Moderate problems	Moderate problems	Slight problems	Slight problems
Pain/discomfort	Moderate problems	Moderate problems	Moderate problems	Moderate problems
Anxiety/depression	Moderate problems	Slight problems	No problem	Slight problems

Ethical considerations

Written informed consent for publication of this case report was obtained from the patient. The patient’s data were anonymized to ensure privacy.

## Discussion

In the present case, numbness and pain caused by CIPN were the main barriers to returning to work. However, through combined exercise therapy and AMF therapy, improvements in physical function and pain relief were achieved, ultimately enabling the patient to return to his occupation.

While CIPN predominantly manifests as sensory axonal neuropathy, it may also affect motor and autonomic functions. Prolonged symptoms can significantly impair physical function and QOL, potentially interfering with continued employment [1,2]. In the present case, the patient’s symptoms persisted for over a year, resulting in significant dysfunction in the hands and lower limbs. Given that the patient’s occupation required manual dexterity and endurance, both sensory and motor impairments needed to be addressed.

Although the patient was taking duloxetine during rehabilitation, adequate pain relief and functional improvement were not achieved. The patient had a history of depression and had been taking duloxetine hydrochloride for an extended period, including during rehabilitation. However, adequate pain relief and functional improvement were not achieved, suggesting that the pharmacological effect of duloxetine was limited in this case. Based on previous studies reporting that exercise therapy can alleviate CIPN symptoms and that combining it with electrical stimulation may enhance therapeutic effects, a physical therapy intervention was considered with the aim of symptom relief and facilitating return to work.

Previous studies have shown that interventions combining strength and aerobic exercises with sensorimotor training are effective in managing CIPN symptoms [4]. In the present case, such an approach improved both upper and lower limb functions, as reflected by an enhancement in QuickDASH scores. A previous study involving breast cancer survivors reported the minimal clinically important difference (MCID) for the 6MWT to be approximately 41 m [17]. In the present case, an improvement exceeding this MCID was observed within one month of initiating physical therapy. Furthermore, a gradual increase in walking distance was noted over time, with an improvement of approximately 170 m at three months compared to baseline, suggesting a clinically meaningful enhancement. Regarding the QuickDASH, prior research has identified the MCID as 15.91, and a comparable improvement was observed in this case [18]. These findings suggest that exercise may have a beneficial effect on physical function in this patient. This aligns with prior findings suggesting that exercise therapy can ameliorate physical function even in the presence of CIPN.

Previous research also reported that combining exercise with electrical stimulation can reduce CIPN symptoms [5]. While transcutaneous electrical nerve stimulation is commonly used for pain management, evidence regarding AMF therapy for sensory symptoms is limited [19]. However, in clinical practice, some patients report discomfort with electrical stimulation. Given that this patient had long-standing numbness and pain, there was concern that he might develop an aversive response to such stimulation. In contrast, AMF therapy delivers magnetic field exposure to alleviate pain and is generally associated with minimal discomfort compared to electrical stimulation. Moreover, AMF therapy has been reported not only to activate descending pain inhibitory pathways via serotonin, norepinephrine, and endogenous opioid mechanisms, but also to exert neuroprotective effects through upregulation of nerve growth factor, contributing to the modulation of neuropathic damage [7-9]. Based on these considerations, AMF therapy was selected over electrical stimulation in this case. As a result, AMF therapy was continued for three months, and its combination with exercise therapy may have contributed to pain relief. In the present case, although the PNQ scores showed some improvement, moderate symptoms remained, making it difficult to draw conclusions regarding AMF’s effectiveness for sensory neuropathy. It is generally known that symptoms may worsen a few months after oxaliplatin administration due to the coasting phenomenon. However, in this case, considering that physical therapy was initiated approximately one year after the final administration of oxaliplatin, spontaneous recovery from the coasting phenomenon alone is unlikely to account for the observed symptom improvement. However, the recurrence of pain during the temporary suspension of AMF therapy and its subsequent improvement upon resumption suggest that AMF therapy may play a role in the management of neuropathic pain. Although this is a single-case report and the possibility of a placebo effect cannot be excluded, the achievement of pain relief is considered meaningful. Previous studies have shown that in patients with CIPN, exercise interventions lasting from 10 to 36 weeks can lead to sustained improvements in physical function and QOL [3,4]. In the present case, an intervention over a period of eight weeks that included both exercise therapy and AMF therapy resulted in improvements in physical function and reduction of pain, which eventually enabled the patient to return to work. We believe that the choice to include AMF therapy, in addition to exercise therapy, played a key role in this outcome. AMF therapy is known to cause little discomfort and is expected to have neuroprotective effects. In this case, the low discomfort associated with AMF therapy may have contributed to improving the feasibility of the intervention. These factors may have promoted better pain control, increased participation in rehabilitation, and ultimately supported the patient in returning to work within two months.

Despite improvements in physical function in the present case, the patient’s QOL scores did not show marked changes. Previous research suggests that optimism may contribute to improved QOL in patients with CIPN; however, the patient in the present case had a history of depression, which may have hindered improvements in his psychological well-being [20]. Thus, future care for patients with CIPN should incorporate not only physical and electrotherapies but also psychological support when needed.

The combination of exercise therapy and AMF therapy in this case contributed to the alleviation of neuropathic pain caused by CIPN, enabling active participation in rehabilitation and subsequent functional improvement. This suggests that multidisciplinary rehabilitation approaches may play a key role in supporting return to work in patients with CIPN.

Limitations and future perspectives

This report describes a single case, which limits the ability to generalize the findings or to determine whether the observed effects resulted from a synergistic interaction between exercise therapy and AMF therapy. Moreover, although pain worsened following the discontinuation of AMF therapy, detailed assessments of physical function during that period were not performed. Additionally, objective diagnostic tools were not employed to assess CIPN or sensory function, making the findings reliant on subjective evaluations. Psychological factors may have influenced the patient’s symptoms; however, the assessment was limited to the anxiety/depression dimension of the EQ-5D-5L.

Future research should include larger sample sizes, use objective measurement tools, integrate comprehensive psychological assessments, and investigate the long-term effects of these interventions. These steps are essential for elucidating the mechanisms and effectiveness of exercise and AMF therapies in treating CIPN.

## Conclusions

The present case suggests the potential benefit of combining exercise therapy with AMF therapy in addressing functional limitations and neuropathic pain in a cancer survivor with CIPN. The patient experienced meaningful recovery and was able to return to work within two months, indicating that such non-pharmacological interventions may support occupational reintegration, particularly when standard treatments are insufficient.

While this report is based on a single case, the clinical improvements observed highlight the need for further investigation into the potential utility of AMF therapy as an adjunct in the rehabilitation of CIPN. Developing individualized programs that integrate physical therapy with emerging modalities may be beneficial in addressing the complex needs of cancer survivors experiencing persistent treatment-related symptoms.
